# Immune response profile of caruncular and trophoblast cell lines infected by high- (Nc-Spain7) and low-virulence (Nc-Spain1H) isolates of *Neospora caninum*

**DOI:** 10.1186/s13071-019-3466-z

**Published:** 2019-05-08

**Authors:** Laura Jiménez-Pelayo, Marta García-Sánchez, Javier Regidor-Cerrillo, Pilar Horcajo, Esther Collantes-Fernández, Mercedes Gómez-Bautista, Nina Hambruch, Christiane Pfarrer, Luis Miguel Ortega-Mora

**Affiliations:** 1 SALUVET, Animal Health Department, Complutense University of Madrid, Ciudad Universitaria s/n, 28040 Madrid, Spain; 2 Department of Anatomy, University of Veterinary Medicine Hannover, Bischofsholer Damm 15, 30173 Hannover, Germany

**Keywords:** *Neospora caninum*, Cattle, Immune response, Placenta, Trophoblast, Caruncle, Isolates, Virulence, Cytokines

## Abstract

**Background:**

Bovine neosporosis, one of the main causes of reproductive failure in cattle worldwide, poses a challenge for the immune system of pregnant cows. Changes in the Th-1/Th-2 balance in the placenta during gestation have been associated with abortion. Cotyledon and caruncle cell layers form the maternal-foetal interface in the placenta and are able to recognize and induce immune responses against *Neospora caninum* among other pathogens. The objective of the present work was to elucidate the immunomodulation produced by high- (Nc-Spain7) and low-virulence (Nc-Spain1H) isolates of *N. caninum* in bovine trophoblast (F3) and caruncular cells (BCEC-1) at early and late points after infection. Variations in the mRNA expression levels of toll-like receptor-2 (TLR-2), Th1 and Th2 cytokines (IL-4, IL-10, IL-8, IL-6, IL-12p40, IL-17, IFN-γ, TGF-β1, TNF-α), and endothelial adhesion molecules (ICAM-1 and VCAM-1) were investigated by RT-qPCR, and protein variations in culture supernatants were investigated by ELISA.

**Results:**

A similar pattern of modulation was found in both cell lines. The most upregulated cytokines in infected cells were pro-inflammatory TNF-α (*P* < 0.05–0.0001) and IL-8 (*P* < 0.05–0.001) whereas regulatory IL-6 (*P* < 0.05–0.001) and TGF-β1 (*P* < 0.05–0.001) were downregulated in both cell lines. The measurement of secreted IL-6, IL-8 and TNF-α confirmed the mRNA expression level results. Differences between isolates were found in the mRNA expression levels of TLR-2 (*P* < 0.05) in both cell lines and in the mRNA expression levels (*P* < 0.05) and protein secretion of TNF-α (*P* < 0.05), which were higher in the trophoblast cell line (F3) infected with the low-virulence isolate Nc-Spain1H.

**Conclusions:**

*Neospora caninum* infection is shown to favor a pro-inflammatory response in placental target cells in vitro. In addition, significant immunomodulation differences were observed between high- and low-virulence isolates, which would partially explain the differences in virulence.

**Electronic supplementary material:**

The online version of this article (10.1186/s13071-019-3466-z) contains supplementary material, which is available to authorized users.

## Background

Bovine neosporosis is one of the main transmissible causes of abortion in cattle worldwide [1–3]. The etiological agent of bovine neosporosis is *Neospora caninum*, an obligate intracellular parasite closely related to the zoonotic agent *Toxoplasma gondii*. Transplacental transmission is the main route of transmission in cattle [4] and the placenta can play a key role in the pathogenesis of bovine neosporosis [5, 6]. The direct damage produced by the multiplication of the parasite in placental and foetal tissues has been proposed as one of the possible causes of abortion observed during *N. caninum* infections. Importantly, the placenta is considered to be an immune regulatory organ since it acts as a modulator of foetal and maternal immune responses. In fact, an immune-mediated pathogenesis has also been suggested as a possible cause of abortion [7]. It has been shown that the multiplication of the parasite in the placenta alters the immunological balance at the maternal-foetal interface with an increase of local pro-inflammatory IFN-γ, IL-12p40 and TNF-α cytokines which could compromise the gestation, together with an increase in IL-4 and IL-10 levels [8, 9], which avoids the immunological rejection of the foetus but favours the multiplication and vertical transmission of the parasite [5, 10]. Trophoblast and caruncular cells are able to recognize pathogens and secrete cytokines and chemokines that recruit immune cells in the damaged area [11–13]. Thus, both cell types play a fundamental role in the initiation of innate immune responses at the placental level as well as in the development of an adaptative immune response for the pregnant dam and foetus.

Previous in vivo studies have shown the influence of the isolate on the dynamics and outcome of the infection in pregnant bovine models and in the cytokine profiles induced during the infection ([9, 14–16], Jiménez-Pelayo et al. unpublished data). To date, only one recent study has utilized an in vitro model consisting of immortalized bovine trophoblasts (F3) from the foetus and caruncular cells (BCEC-1) from the dam. The aim of the study was to elucidate the interactions between tachyzoites and the host cells that resemble the maternal-foetal interface of the bovine placentome while also taking into account the influence of the isolate. Maternal cells, where both isolates showed similar phenotypic traits, presented higher resistance to the infection than trophoblast cells, where the high- (Nc-Spain7) and the low-virulence (Nc-Spain1H) isolates showed marked differences in proliferation [17].

However, the interactions between the parasite and the placental target cells from an immunological point of view have not been investigated in vitro until now. Thus, the objective of the present study was to compare the immune response profiles of the bovine placental cells in vitro after the infection with two *N. caninum* isolates of different virulence. Messenger RNA expression levels of TLR-2, pro-inflammatory cytokines IL-8, IL-12p40, IL-17, IFN-γ, TNF-α, anti-inflammatory/regulatory cytokines TGF-β1, IL-4, IL-6 and IL-10 as well as ICAM-1 and VCAM-1 endothelial adhesion molecules were determined at 4 and 24 hours post-infection (hpi) in maternal caruncular (BCEC-1) and foetal trophoblast (F3) cell cultures and protein secretion was assessed in culture supernatants by ELISA.

## Results

### Expression profile of TLR-2

**Fig. 1:**
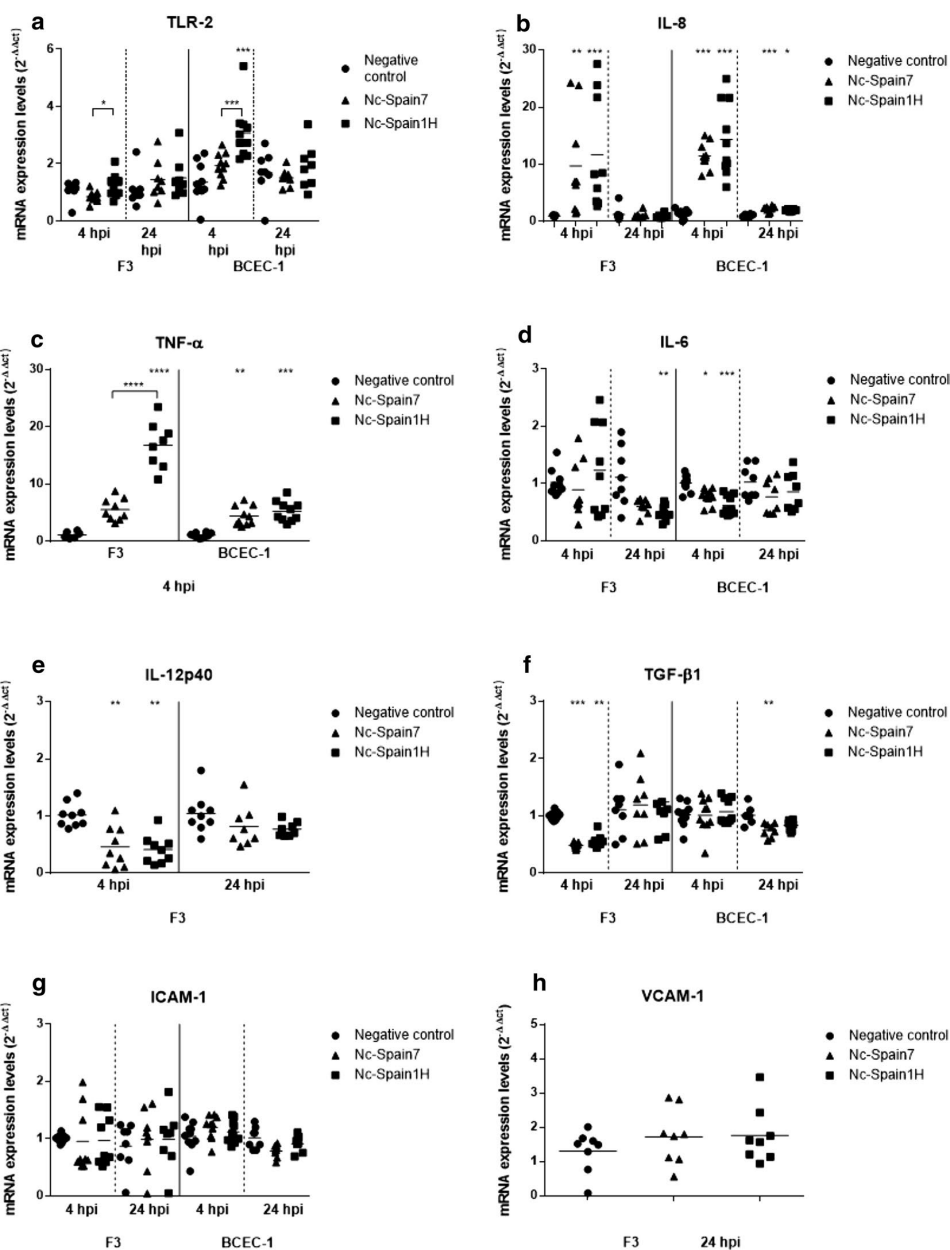
TLR-2, IL-8, TNF-α, IL-6, IL-12p40, TGF-β1, ICAM-1 and VCAM-1 transcript expression. Scatter-plot graphs of relative mRNA expression levels (as x-fold change) of TLR-2 (**a**), IL-8 (**b**), TNF-α (**c**), IL-6 (**d**), IL-12p40 (**e**), TGF-β1 (**f**), ICAM-1 (**g**) and VCAM-1 (**h**) in F3 and BCEC-1 cell cultures at 4 and 24 hpi with Nc-Spain7 and Nc-Spain1H isolates. Data are represented as individual points. Horizontal lines represent median values for each group. ****P < 0.0001, ***P < 0.001, **P < 0.01, *P < 0.05. Unbracketed symbols represent differences with respect to the control group, while significant differences between isolates are denoted by horizontal square brackets

Our results showed that *N. caninum* infection for 4 h in BCEC-1 cells resulted in a significant upregulation of TLR-2 expression in Nc-Spain1H-infected cells compared with that of negative control cells (Kruskal–Wallis H-test followed by Dunnʼs multiple comparison test: *χ*^2^ = 16.2, *df* = 2, *P* = 0.0001) and with that of BCEC-1 cells infected with the high-virulence isolate Nc-Spain7 (Mann–Whitney U-test: *U*_(8)_ = 8, *Z* = 2.591, *P* = 0.0007). In F3 cultures, statistical significance was not found at either 4 or 24 hpi between infected groups and the control group. However, comparing both isolates, lower expression of TLR-2 was found in the F3 cultures infected with Nc-Spain7 than in the F3 cultures infected with the low-virulence isolate Nc-Spain1H at 4 hpi (Mann–Whitney U-test: *U*_(8)_ = 16, *Z* = 2.287, *P* = 0.0315) (Fig. 1a).

### Pro-inflammatory and regulatory cytokine modulation

**Fig. 2:**
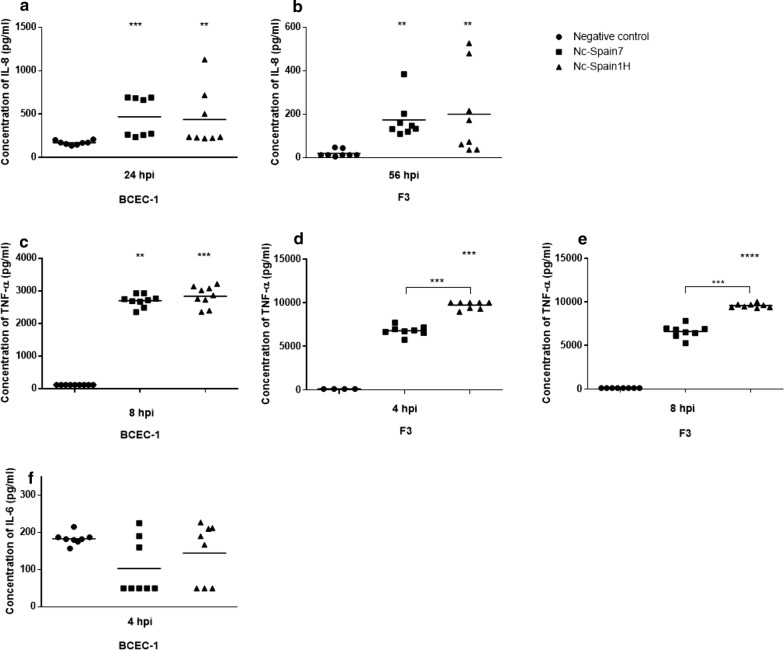
IL-8, TNF-α and IL-6 secretion levels in culture supernatants. Scatter-plot graphs representing the concentration of IL-8 (pg/ml) in BCEC-1 (**a**) and F3 (**b**) supernatants infected with Nc-Spain7 and Nc-Spain1H at 24 and 56 hpi, respectively, the concentration of TNF-α (pg/ml) in the BCEC-1 supernatants at 8 hpi (**c**) and in the F3 supernatants at 4 hpi (**d**) and 8 hpi (**e**), and the concentration of IL-6 (pg/ml) in the BCEC-1 supernatants at 4 hpi (**f**). Data are represented as individual points. Horizontal lines represent median values for each group. ****P < 0.0001, ***P < 0.001, **P < 0.01, *P < 0.05. Unbracketed symbols represent differences with respect to the control group, while significant differences between isolates are denoted by horizontal square brackets

The pro-inflammatory cytokines IL-8 (Kruskal–Wallis H-test: *χ*^2^ = 19.52, *df* = 2, *P* < 0.0001 in BCEC-1 and *χ*^2^ = 17.56, *df* = 2, *P* = 0.0002 in F3) and TNF-α (Kruskal–Wallis H-test: *χ*^2^ = 19.73, *df* = 2, *P* < 0.0001 in BCEC-1 and *χ*^2^ = 19.4, *df* = 2, *P* < 0.0001 in F3) were upregulated in both cell types at 4 hpi compared to the respective control groups (Fig. 1b, c). At 24 hpi, IL-8 expression was still increased in BCEC-1 cells infected by both isolates (Kruskal–Wallis H-test followed by Dunnʼs multiple comparison test: *χ*^2^ = 16.63, *df* = 2, *P* = 0.0003 and *χ*^2^ = 10.19, *df* = 2, *P* = 0.0117 for Nc-Spain7 and Nc-Spain1H, respectively); however, the increment of IL-8 had disappeared at 24 hpi in F3-infected cells with respect to the control group. When both isolates were compared, Nc-Spain1H induced a higher expression of TNF-α than the high-virulence isolate Nc-Spain7 at 4 hpi in F3 cells (Mann–Whitney U-test: *U*_(8)_ = 0, *Z* = 2.579, *P* < 0.0001). Protein levels of the pro-inflammatory cytokines IL-8 and TNF-α were also investigated in the supernatant of control and infected cultures at different time-points post-infection. A higher secretion of IL-8 was found for both isolates in BCEC-1 cells at 24 hpi (Kruskal–Wallis H-test: *χ*^2^ = 15.87, *df* = 2, *P* = 0.0004) and in F3 cells at 56 hpi (Kruskal–Wallis H-test: *χ*^2^ = 13.74, *df* = 2, *P* = 0.001) with respect to the control group (Fig. 2a, b). Secretion of TNF-α was higher in BCEC-1 cells infected by both isolates (Kruskal–Wallis H-test: *χ*^2^ = 18.9, *df* = 2, *P* < 0.0001; Fig. 2c) and in F3 cells infected by Nc-Spain1H (Kruskal–Wallis H-test followed by Dunnʼs multiple comparison test: *χ*^2^ = 16*, df* = 2*, P* < 0.0001) at 8 hpi, although an earlier secretion of TNF-α was also found in F3 cells infected by Nc-Spain1H (*χ*^2^ = 14*, df* = 2*, P* = 0.0003) at 4 hpi (Fig. 2d, e). As observed with the TNF-α mRNA expression, Nc-Spain1H induced a higher secretion of TNF-α than did the high-virulence isolate Nc-Spain7 at 4 hpi (Mann–Whitney U-test: *U*_(8)_ = 0*, Z* = 2.736*, P* = 0.0002) and at 8 hpi (*U*_(8)_ = 0*, Z* = 2.305*, P* = 0.0002) in the F3 cultures (Fig. 2d, e).

The expression levels of other important cytokines associated with *N. caninum* infection, such as IL-12p40 and IL-6 (Fig. 1d, e), were modified in placental cells after parasite infection. Specifically, IL-6 levels were downregulated in BCEC-1 infected by Nc-Spain1H and Nc-Spain7 at 4 hpi (Kruskal–Wallis H-test: *χ*^2^ = 16.08, *df* = 2, *P* = 0.0003) and F3 cultures infected by Nc-Spain1H at 24 hpi (*χ*^2^ = 10.5, *df* = 2, *P* = 0.0052). IL-12p40 was also downregulated but only in infected F3 cultures at 4 hpi (*χ*^2^ = 12.99, *df* = 2, *P* = 0.0015). Differences between isolates in the modulation of IL-6 and IL-12p40 were not found. In addition, we observed that the caruncular cell layer did not express IL-12p40 mRNA at any time point. The decrease in the expression of IL-6 observed in infected BCEC-1 cells was confirmed by the decrease in the secretion levels of that protein found in the supernatants from BCEC-1 cultures infected with both isolates at 4 hpi, although statistically significant differences were not found (Kruskal–Wallis H-test: *χ*^2^ = 2.765*, df* = 2*, P* = 0.251), probably because of the high deviation between samples (Fig. 2f).

Finally, pro-inflammatory IL-17 and IFN-γ responses were not detected in any cell lines at 4 nor at 24 hpi.

We also studied the mRNA levels of the anti-inflammatory cytokines TGF-β1, IL-4 and IL-10. Remarkably, we observed a decrease in the expression levels of TGF-β1 in both cell lines infected with both isolates. Specifically, a decrease was observed at 4 hpi in F3 cultures (Kruskal–Wallis H-test: *χ*^2^ = 18.44, *df* = 2, *P* < 0.0001) and at 24 hpi in BCEC-1 cultures (*χ*^2^ = 12.02, *df* = 2, *P* = 0.0025) (Fig. 1f). No differences between isolates were observed in the mRNA expression levels of TGF-β1. There was not detection in bovine placental cells of the anti-inflammatory cytokine IL-4 or the regulatory cytokine IL-10 at 4 and 24 hpi.

### Endothelial adhesion molecule (ICAM-1 and VCAM-1) expression

The adhesion molecule ICAM-1 was expressed by both cell lines at 4 and 24 hpi. However, only a slight decrease in the mRNA expression levels of ICAM-1 was observed in the BCEC-1 cultures infected with Nc-Spain7 at 24 hpi compared to the control group although statistical significance was not found (Kruskal–Wallis H-test: *χ*^2^ = 5.894, *df* = 2, *P* = 0.0525) (Fig. 1g). VCAM-1 expression was detected only in the F3 cultures at 24 hpi, but differences between the infected and the control groups were not found in this culture at this time point (Fig. 1h).

Results of mRNA expression levels and protein secretion from statistical tests are reported in Additional file 1: Tables S1 and S2, respectively.

## Discussion

Transmission of *N. caninum* across the placenta makes this organ key in the pathogenesis of bovine neosporosis. Innate immune signalling is crucial at the maternal-foetal interface, where vertical transmission of pathogens to the foetus can have profound pathological outcomes. Trophoblasts and other cell types within the placenta may also be involved in the physiological protection of the placenta [18]. Trophoblast cells have been shown to respond to some infections by producing pro-inflammatory cytokines and chemokines and endometrial or decidual cells can produce and secrete a variety of cytokines, participating in the attraction and activation of immune effector cells [19, 20]. However, these innate immune mechanisms are unexplored at the maternal-foetal interface during *N. caninum* infection in pregnant cattle [21].

The expression of TLRs has been described in trophoblasts and other cell types within the placenta [22]. Specifically, TLR-2 was overexpressed in bovine trophoblast cell cultures at 8 hpi [23] and TLR-3, 7 and 8 have been implicated in *N. caninum* recognition in the bovine placenta [21, 24]. In our study, differential activation of TLR-2 in the F3 and BCEC-1 cultures was observed. An upregulation of TLR-2 was found in BCEC-1-infected cultures, especially in those infected with the low-virulence isolate Nc-Spain1H. The caruncular part of the placentome showed a higher expression of several TLRs, suggesting that the initial recognition of *N. caninum* at the placental level would occur in the maternal side of placenta [21]. Taking into account the data shown in Jiménez-Pelayo et al. [17], which confirm the higher proliferation of *N. caninum* in trophoblast cells, an important role of placental TLR-2 in the immune response against *N. caninum* seems plausible. TLR activation is crucial for initiating the innate immune responses responsible for the elimination of intracellular parasites such as *N. caninum*, and the signalling pathway activated by TLR-2 leads to an increase in the transcription factors NF-κβ and AP-1, which trigger the synthesis of pro-inflammatory cytokines (TNF-α, IL-6, IL-12 and IL-1β) and chemokines (IL-8, RANTES) [25].

Despite differential modulation of host TLR-2, both cell types presented a similar variation in the IL-6, TNF-α and IL-8 expression levels in infected cultures. The pro-inflammatory IL-8 and TNF-α cytokines were upregulated, and secretion of the proteins in the supernatants of both cell lines was also detected by ELISA. IL-8, a cytokine with neutrophil chemotactic/activating and T-cell chemotactic activity both in vivo and in vitro, is important in the recruitment of leukocytes to the endometrium and may be a potential mediator of placental macrophage infiltration [26], which might help to eliminate the parasite. IL-8 upregulation has already been observed in bovine umbilical vein endothelial cells (BUVECs) infected by *T. gondii* and *N. caninum* [27] as well as in bovine trophoblastic cells and placentomes from cows infected with *Brucella abortus* [28]. TNF-α is an inflammatory cytokine whose expression has also been described for epithelial cells [29]. TNF-α is expressed in all cell types of the trophoblastic lineage and provokes a variety of biological effects on placental and endometrial cell types [29]. In addition, TNF-α, through the NF-κβ signalling pathway, coordinates the inflammatory response via the induction of other cytokines (IL-1 and IL-6) and chemokines (IL-8) and via the upregulation of adhesion molecules (ICAM-1 and VCAM-1) [30, 31], playing a role favouring protective immunity in infectious diseases [32]. There are several lines of experimental evidence indicating that TNF-α plays a role not only in immunity to *N. caninum* but also in the immunopathology of neosporosis. TNF-α expression and secretion may reduce the parasite presence in the placenta by inhibiting the intracellular multiplication of the parasite [33] and participating in parasite proliferation control mechanisms [34]; however, TNF-α expression is detrimental to pregnancy maintenance [8].

IL-6 expression levels were diminished in infected cultures of BCEC-1 and F3 at 4 and 24 hpi, respectively. The classification of IL-6 as Th1 or Th2 has been considered controversial since it can have characteristics of both depending on the dose, the cellular source and the gestational stage studied [35]. Currently, the presence of IL-6 displaces the Th1/Th2 balance towards a Th2 response [36]. However, in vivo models of *N. caninum* infection have shown IL-6 upregulation [37, 38, Jiménez-Pelayo et al. unpublished data]. This response pattern may be related to a protective action that protects the foetus and allows gestation even if the animals are born infected [10]. The decrease in IL-6 observed could be explained by the following: (i) IL-6 expression levels were affected by the high antigenic dose administered (MOI 10), resulting in downregulation [39]; (ii) the time points were not adequate for detecting the peak of IL-6 expression, and the observed decrease may be the consequence of the rapid reduction in IL-6 expression after a peak of expression [40–42]; or (iii) other cell types are implicated in the upregulation of IL-6 that was observed in vivo.

The anti-inflammatory cytokine TGF-β1 was also found to be downregulated in F3 and BCEC-1 cultures at 4 and 24 hpi, respectively. Several members of the TGF-β superfamily have been suggested to regulate trophoblast cell functions, and their dysregulation has been implicated in pregnancy-associated diseases. TGF-β1 is crucial in neutralizing the inflammatory responses induced by Th1-type cytokines [5]. This effect has already been observed in previous works where the reduction of TGF-β1 was shown to be beneficial for controlling *N. caninum* growth but detrimental for the adequate maintenance of pregnancy [38, 43].

The reduction of pro-inflammatory IL-12p40 observed in trophoblast cells, together with the lack of expression of IL-12p40 in BCEC-1 cultures, disagrees with the results of previous experimental infections [8, 9, 38, 44, 45]. Similarly, the lack of expression of pro-inflammatory IFN-γ, essential for controlling parasite infection [1, 46], and anti-inflammatory IL-4 and IL-10, related to placental protection during *N. caninum* infections [8, 9], lead us to hypothesize that the upregulation of IL-12p40, IFN-γ, IL-4 and IL-10 observed in vivo could be attributed to immune cells present in the placenta, such as dendritic cells, NK cells or macrophages. Therefore, trophoblast and/or caruncular cells would not be responsible for the direct production of these cytokines, although the assayed time points may not have been appropriate for their detection.

Finally, ICAM-1 and VCAM-1 expression were not modulated by the parasite infection. These adhesion molecules participate in the recruitment of inflammatory immune cells [47] and promote the adherence of monocytes to endothelial cells [48]. The upregulation of ICAM-1 has been observed in in vitro infections with apicomplexan parasites [27, 49, 50]. The absence of modulation observed in this work may be explained by differences in the timing of the expression of ICAM-1 and VCAM-1 [27, 49, 50] or by the lack of stimuli such as the acute-phase protein C-reactive protein (CRP), which is produced by the liver in response to IL-6 [51].

As mentioned above, the parasite isolate is a key factor in the outcome of the infection. In general, differences in the modulation between high- and low-virulence isolates were not remarkable in trophoblast or caruncular cells, with the exception of the mRNA expression levels of TLR-2 and TNF-α. TLR-2 levels were more upregulated by Nc-Spain1H infection than by Nc-Spain7 infection in both cell lines, which led us to hypothesize that the high-virulence isolate would activate less of the TLR recognition system, reducing the immune responses triggered by TLR-2. The inhibition of the TLRs implicated in the recognition of *Trypanosoma cruzi* and *T. gondii* in HPCVE increased the parasite burden and, importantly, TLR-2 inhibition prevented the secretion of IL-6 and IL-10, increasing parasite damage [52, 53]. The low-virulence isolate Nc-Spain1H activates the expression of TLR-2, starting an inflammatory response, which may be the cause of the lower proliferation of this isolate [17, 54] in addition to being one of the causes that explains the higher levels of TNF-α in Nc-Spain1H-infected cells, especially in trophoblast cells. Our results suggest that differential activation of the TLRs by the isolates of differing virulences should be subject to future research since they may be responsible for the biological differences observed both in vitro and in vivo.

The low-virulence isolate Nc-Spain1H also induced higher expression of TNF-α in F3. A higher TNF-α response may more efficiently control the proliferation of Nc-Spain1H in F3 cultures, which could explain the observations made by Jiménez-Pelayo et al. [17] where lower proliferation of Nc-Spain1H was observed in these cells. The lower expression of TNF-α observed during the early stage of infection of trophoblasts with the high-virulence isolate Nc-Spain7 supports the hypothesis that this isolate may modify by yet unknown mechanisms the pro-inflammatory response by trophoblast cells. However, how Nc-Spain7 is able to evade the immune response and maintain lower levels of TNF-α expression in F3 remains unknown. On the other hand, these results suggest that pro-inflammatory cytokines such as TNF-α could have a minor impact in placental damage than postulated in previous works [8, 55], but other mechanisms should be implicated in placental damage in vivo and the occurrence of abortion, such as the high multiplication ability showed by the high-virulence isolate Nc-Spain7 [17].

## Conclusions

The results presented in this manuscript suggest that placental cells participate in the innate immune response at the maternal-foetal interface via a rapid pro-inflammatory response characterized by the overexpression of IL-8 and TNF-α and the downregulation of TGF-β1 and IL-6. Slight differences were detected when the immunomodulatory response induced by the high and low virulent *N. caninum* isolates was compared. The higher expression of TLR-2 in the F3 and BCEC-1 cells and the TNF-α in F3 cells infected with the low-virulence isolate Nc-Spain1H may indicate a higher stimulation of the immune response by this isolate or a higher immunomodulation of Nc-Spain7, which could explain the biological differences observed in vitro and in vivo. F3 and BCEC-1 cultures seem to be a good tool for the study of the TLR activation mechanisms by *N. caninum.* Finally, we observed that cytokines such as IFN-γ, IL-4 or IL-10, which are commonly upregulated in the placenta after *N. caninum* infection, are not expressed in F3 and BCEC-1 cells; we conclude that the trophoblast and caruncular epithelial cells are not implicated in the production of these cytokines in the placenta or that other pathways/cells/molecules are needed for their production.

## Methods

### Parasites and cell cultures

A full description of the Nc-Spain1H and Nc-Spain7 parasites and cell cultures of bovine caruncular epithelial (BCEC-1) and bovine trophoblast cells (F3) is provided in a previous report [17]. Briefly, Nc-Spain7 and Nc-Spain1H isolates were obtained from healthy, congenitally infected calves [56, 57] and tachyzoites were maintained in a MARC-145 culture as described previously [54]. The number of culture passages of both *N. caninum* isolates was limited (passages from 9 to 11) to maintain their biological in vivo behavior [58].

The BCEC-1 and F3 cell lines were kindly donated by Dr C. Pfarrer from the University of Veterinary Medicine Hannover and maintained following the protocols described in the literature [59, 60].

### Infection of the cultures, collection and preservation of the samples

BCEC-1 and F3 cells were seeded in 25 cm^2^ culture flasks adjusting the number of cells in order to obtain a confluent monolayer after 24 h of culture. F3 was seeded at 10^6^ cells per flask, whereas BCEC-1 was seeded at a concentration of 2 × 10^6^ cells per flask. Tachyzoites were recovered from MARC-145 cultures when most of the parasites were still inside parasitophorous vacuoles; tachyzoites were purified using disposable PD-10 Desalting Columns (G.E. Healthcare, Amersham, UK) as previously described [54]. The parasite viability was checked by trypan blue exclusion, and the tachyzoites were counted. Multiplicity of infection (MOI) of 8 (8 × 10^6^ tachyzoites in F3 and 16 × 10^6^ tachyzoites in BCEC-1) and 10 (10^7^ tachyzoites in F3 and 2 × 10^7^ tachyzoites in BCEC-1) from the Nc-Spain7 and Nc-Spain1H isolates, respectively, were inoculated into confluent monolayers of F3 and BCEC-1 quickly after collection. Due to the differences observed in the infection rate between isolates [17], different MOIs of each isolate were selected with the aim of obtaining cultures infected with the same quantity of each parasite at 4 and 24 hpi. This way possible differences in the modulation of the mRNA expression levels between isolates could be attributed to differences in their biological behavior and not to the differences in the parasite burden. In addition, cultures were infected with high doses of both parasites to get a high infection of the cultures at 4 and 24 hpi so that the RNA from uninfected cells did not mask possible differences in RNA expression levels induced by the infection. The flasks were incubated at 37 °C until collection of the samples. The supernatants were collected at different time points (4, 8, 24 and 56 hpi) and stored at −80 °C for the detection of proteins by ELISA. The cultures were harvested at 4 or 24 hpi by scraping, centrifugation at 1350×*g* for 15 min at 4 °C and resuspending the pellet in 300 µl of RNAlater^®^ (Qiagen, Hilden, Germany). The samples were stored at −80 °C prior to RNA extraction.

Two independent experiments were carried out and four replicates were obtained in each experiment.

### RNA extraction, reverse transcription and quantitative real-time PCR

The mRNA expression levels of TLR-2, pro-inflammatory cytokines IL-6, IL-8, IL-12p40, IL-17, IFN-γ, TNF-α, anti-inflammatory/regulatory cytokines TGF-β1, IL-4 and IL-10 as well as ICAM-1 and VCAM-1 endothelial adhesion molecules were determined by real-time RT-PCR in the F3 and BCEC-1 cell layers infected with the high-virulence isolate (Nc-Spain7) and the low-virulence isolate (Nc-Spain1H) of *N. caninum* at an early (4 hpi) and a late (24 hpi) time point.

RNA was extracted using a commercial Maxwell^®^ 16 LEV simplyRNA Purification kit (Promega, Madison, WI, USA) following the manufacturer’s recommendations. RNA integrity was checked by 1% agarose gel and RNA concentrations were determined using a NanoPhotometer^®^ spectrophotometer (Implen, Munich, Germany). cDNA was obtained by reverse transcription of 2.5 µg of RNA using the master mix SuperScript^®^ VILO™ cDNA Synthesis kit (Invitrogen, Paisley, UK), which was diluted 1:20 in molecular grade water for the qPCR assays.

The PCRs were performed using 12.5 µl of Power SYBR^®^ Green PCR Master Mix (Applied Biosystems, Foster City, CA, USA), 10 pmol of each primer (except for TLR-2 primers which were used at a concentration of 22.5 pmol) and 5 μl of diluted cDNA samples in an ABI 7300 Real Time PCR System (Applied Biosystems). The primers used for the qPCR reactions are shown in Table 1. β-Actin and GAPDH were used as housekeeping genes, obtaining comparable Ct values for all the samples. For each target gene, a seven-point standard curve was included in each batch of amplifications based on 10-fold serial dilutions starting at 10 ng/µl of plasmid DNA. The relative quantification of the mRNA expression levels (x-fold change in expression) was carried out by the comparative 2^−ΔΔCt^ method [63].

**Table 1:** Sequences of primers used for cytokine real-time PCR (qPCR) and standard curve data

Target^a^	Primer	Primer sequence (5′–3′)	Product size (bp)	*R* ^2 b^	Slope^c^
IFN-γ (NM_174086.1)	QIFN-UP^g^	GATTCAAATTCCGGTGGATG	110	0.994	(−3.47)–(−3.30)
	QIFN-RP^g^	TTCTCTTCCGCTTTCTGAGG			
TNF-α (EU276079.1)	QTNF-UP^g^	CCAGAGGGAAGAGCAGTCC	126	0.998	(−3.39)–(−3.27)
	QTNF-RP^g^	GGAGAGTTGATGTCGGCTAC			
IL-4 (M77120.1)	QIL4-UP^g^	CTGCCCCAAAGAACACAACT	169	0.995	(−3.33)–(−3.54)
	QIL4-RP^g^	GTGCTCGTCTTGGCTTCATT			
IL-6 (X68723.1)	QIL-6-UP^d^	CTGGGTTCAATCAGGCGATT	150	0.999	(−3.22)–(−3.20)
	QIL-6-RP^d^	GGATCTGGATCAGTGTTCTGA			
IL-8 (BC103310.1)	qIL8-Fw^h^	CCACACCTTTCCACCCCAAA	177	0.995	(−3.36)–(−3.23)
	qIL8-Rw^h^	CTTGCTTCTCAGCTCTCTTC			
IL-10 (NM_174088.1)	QIL10-UP^g^	TGCTGGATGACTTTAAGGGTTACC	60	0.999	(−3.27)–(−3.42)
	QIL10-RP^g^	AAAACTGGATCATTTCCGACAAG			
IL-12p40 (NM_174356.1)	QIL12-UP^g^	AGTACACAGTGGAGTGTCAG	157	0.992	(−3.39)–(−3.35)
	QIL12-RP^g^	TTCTTGGGTGGGTCTGGTTT			
IL-17 (NM_001008412.1)	qIL17bov-up^h^	GAACTTCATCTATGTCACTGC	83	0.997	(−3.30)–(−3.18)
	qIL17bov-rev^h^	TGGACTCTGTGGGATGATGA			
TGF-β1 (NM_001009400.1)	QTGF-UP^d^	GGTGGAATACGGCAACAAAA	117	0.999	(−3.60)–(−3.53)
	QTGF-RP^d^	CGAGAGAGCAACACAGGTTC			
TLR-2 (NM_001048231.1)	QTLR2-UP^e^	ACGACGCCTTTGTGTCCTAC	192	0.993	(−3.74)–(−3.38)
	QTLR2-RP^e^	CCGAAAGCACAAAGATGGTT			
ICAM-1 (NM_174348.2)	qICAM-Fw^h^	AGACCTATGTCCTGCCATCG	219	0.994	(−3.34)–(−3.30)
	qICAM-Rw^h^	GGTGCCCTCCTCATTTTCCT			
VCAM (XM_005204079.2)	qVCAM-Fw^h^	GAACTGGAAGTCTACATCTC	128	0.998	(−3.36)–(−3.32)
	qVCAM-Rw^h^	CAGAGAATCCGTGGAGCTGG			
GAPDH (NM_001034034)	GAPDH-F^f^	ATCTCGCTCCTGGAAGATG	227	0.996	(−3.67)–(−3.58)
	GAPDH-R^f^	TCGGAGTGAACGGATTCG			
β-Actin (NM_173979.3)	BACTIN-UP^g^	ACACCGCAACCAGTTCGCCAT	216	0.994	(−3.45)–(−3.36)
	BACT216-RP^g^	GTCAGGATGCCTCTCTTGCT			

^a^NCBI accession numbers are for cDNA sequences used in primer design. Primer annealing was also checked with the *Bos taurus* genomic DNA sequences (http://www.ncbi.nlm.nih.gov/nuccore)

^b^Minimum coefficient of regression (*R*^*2*^) of standard curves for each PCR target in all batches of amplification

^c^Standard curve slopes. Minimum and maximum values for slopes for each PCR target in all batches of amplification

^d^Primer first described by Arraz-Solís et al. [43]

^e^Primer first described by Menzies & Ingham [61]

^f^Primer first described by Puech et al. [62]

^g^Primer first described by Regidor-Cerrillo et al. [9]

^h^Primer described in the present work for the first time

### Measurement of cytokines in supernatants of BCEC-1 and F3 cell cultures by ELISA

Protein concentrations of the cytokines that showed variations in the mRNA expression levels were determined in the culture supernatants at 4, 8, 24 and 56 hpi using commercial ELISA kits. The levels of IL-6, IL-8 and TNF-α cytokines were measured in the supernatants of the BCEC-1 and F3 cells by sandwich ELISAs using a Bovine IL-6 ELISA Reagent kit (ESS0029; Thermo Fisher Scientific, Waltham, MA, USA), Bovine IL-8 (CXCL8) ELISA Development kit (3114-1A-6; Mabtech AB, Stockholm, Sweden) and Bovine TNF-α ELISA kit (EBTNF; Thermo Fisher Scientific) following the manufacturers’ instructions. The sensitivity limits of these assays were 78 pg/ml for IL-6, 25 pg/ml for IL-8 and 100 pg/ml for TNF-α.

### Statistical analysis

TLR, cytokine and endothelial adhesion molecule mRNA expression levels, as well as differences in the protein secretion between infected and control groups, were analysed using the non-parametric Kruskal–Wallis test, followed by Dunn’s multiple comparison test for all pairwise comparisons. In addition, to assess differences between both infected groups a Mann–Whitney test was performed for each molecule analysed. The statistical significance for all the analyses was established with *P* < 0.05. GraphPad Prism v.5.01 software (GraphPad Software, San Diego, CA, USA) was used to perform all statistical analyses and create all the graphical illustrations.

## Additional file

**Additional file 1:Table S1.** Statistical test results for mRNA expression levels. **Table S2.** Statistical test results for protein secretion.

## Abbreviations

BCEC-1: bovine caruncular epithelial cell line 1
F3: bovine placental trophoblast cell line
qPCR: real-time polymerase chain reaction
hpi: hours post-infection
BVD: bovine viral diarrhea
DMEM: Dulbecco’s modified Eagle medium
FCS: foetal calf serum
PBS: phosphate buffered saline
TLR-2: Toll-like Receptor 2
IL: interleukin
IFN: interferon
TGF: transforming growth factor
TNF: tumoral necrosis factor
ICAM: intercellular adhesion molecule
VCAM: vascular cell adhesion molecule
ELISA: enzyme-linked immunosorbent assay
BUVECs: bovine umbilical vein endothelial cells
HPCVE: human placental chorionic villi explants
MOI: multiplicity of infection
